# Occupational inhalation exposure during surface disinfection—exposure assessment based on exposure models compared with measurement data

**DOI:** 10.1038/s41370-023-00633-y

**Published:** 2023-12-25

**Authors:** Lea Anhäuser, Benedikt Piorr, Mario Arnone, Wolfgang Wegscheider, Johannes Gerding

**Affiliations:** 1 German Social Accident Insurance Institution for the Health and Welfare Services (BGW), Department for Occupational Medicine, Hazardous Substances and Public Health, Pappelallee 33/35/37, 22089 Hamburg, Germany; 2 Federal Institute for Occupational Safety and Health (BAuA), Unit Exposure Assessment Biocides, Friedrich-Henkel-Weg 1-25, 44149 Dortmund, Germany; 3 Institute for Occupational Safety and Health (IFA) of the German Social Accident Insurance (DGUV), Section Exposure Monitoring-MGU, Alte Heerstrasse 111, 53757 Sankt Augustin, Germany

**Keywords:** Surface disinfection, Inhalation exposure, Exposure assessment, Exposure modelling, Measurement data, Model quality

## Abstract

**Background:**

For healthcare workers, surface disinfections are daily routine tasks. An assessment of the inhalation exposure to hazardous substances, in this case the disinfectant´s active ingredients, is necessary to ensure workers safety. However, deciding which exposure model is best for exposure assessment remains difficult.

**Objective:**

The aim of the study was to evaluate the applicability of different exposure models for disinfection of small surfaces in healthcare settings.

**Methods:**

Measurements of the air concentration of active ingredients in disinfectants (ethanol, formaldehyde, glutaraldehyde, hydrogen peroxide, peroxyacetic acid) together with other exposure parameters were recorded in a test chamber. The measurements were performed using personal and stationary air sampling. In addition, exposure modelling was performed using three deterministic models (unsteady 1-zone, ConsExpo and 2-component) and one modifying-factor model (Stoffenmanager®). Their estimates were compared with the measured values using various methods to assess model quality (like accuracy and level of conservatism).

**Results:**

The deterministic models showed overestimation predominantly in the range of two- to fivefold relative to the measured data and high conservatism for all active ingredients of disinfectants with the exception of ethanol. With Stoffenmanager® an exposure distribution was estimated for ethanol, which was in good accordance with the measured data.

**Impact statement:**

To date, workplace exposure assessments often involve expensive and time consuming air measurements. Reliable exposure models can be used to assess occupational inhalation exposure to hazardous substances, in this case surface disinfectants. This study describes the applicability of three deterministic and one modifying-factor model for disinfection of small surfaces in healthcare settings, in direct comparison to measurements performed and will facilitate future exposure assessments at these workplaces.

## Introduction

Disinfection activities are frequently conducted in hygiene-critical areas, especially in healthcare settings ranging from large hospitals and nursing homes to smaller facilities such as doctors’ offices or outpatient care services. In addition to other disinfection activities (e.g. hand disinfection or disinfection of medical devices) or patient treatment and care, surface disinfection is a key daily activity for healthcare workers. However, surface disinfection is also conducted in social institutions such as kindergartens, educational or youth facilities as well as in the food-processing industry.

Products used for surface disinfection in healthcare settings may contain active ingredients that are non-volatile or volatile at room temperature. Examples of non-volatile active ingredients are quaternary ammonium compounds, alkylamines or biguanides [1, 2]. Volatile ingredients are usually alcohols such as ethanol and 2-propanol, aldehydes such as formaldehyde or glutaraldehyde, peroxides such as hydrogen peroxide and peroxyacetic acid or active chlorine releasing agents. The resulting inhalation exposure of healthcare workers may pose a health risk due to the frequent use of disinfectants containing the above-mentioned volatile ingredients, leading to respiratory diseases such as COPD [3–6] or asthma [7, 8]. The disinfectant active ingredients can also cause skin diseases such as irritant-toxic and allergic contact dermatitis [9–11].

Before starting a disinfection activity, the employer has to identify and assess the workers exposure to the disinfectants active ingredients in the selected product. The European ‘Guidance on risk assessment at work’ has described this approach of safe working at the workplace since 1996 [12]. For the assessment of the inhalation exposure during an activity with hazardous substances either measurements or non-measurement methods are suitable.

Measured or modelled concentrations of a hazardous substance in the air, therefore serve as the unit for inhalation exposure assessment. For the implementation of workplace measurements, there is also the European standard DIN EN 689 [13] or, in Germany additionally the ‘Technical Rules for Hazardous Substances’ [14, 15]. Non-measurement methods include, for example, exposure models. These models are not only used in the registration procedure under REACH or the authorisation of biocidal products according to the Biocidal Product Regulation (BPR) [16–18], but also in workplace-specific exposure assessment when workplace measurements are not feasible or available [13, 15]. With the result of the exposure assessment, appropriate measures to protect workers from exposure have to be established and, if necessary, a less hazardous disinfectant is to be selected.

Two different approaches are commonly used for exposure modelling: The modifying-factor models based on multiplicative exposure-determining factors and the deterministic physico-chemical models based on mass-balances [19–21]. Examples include Stoffenmanager® [22–25] and ART (Advanced REACH Tool) [26] as modifying-factor models, and ConsExpo [27] as a deterministic model. To date, the various exposure models have not been sufficiently evaluated and validated, making it difficult to decide which model suites best for exposure assessment for a hazardous substance in a specific workplace [21, 28]. Various analysis methods were used to learn more about the performance of the models, e.g., by comparing them with measured data in terms of accuracy or conservatism [29, 30].

Studies comparing modelled data with measured data in occupational exposure scenarios often rely on independent measurement data from real workplaces available in scientific literature or other databases [31–35]. Another option is the measurement of workers exposures in combination with the exposure modelling for the same specific workplace. Thereby, detailed information on the workplace conditions and work process is available, which provides more precise input parameters for the exposure models. Such projects have been conducted, for example, for spray paint exposure with toluene in industry or exposure of the anaesthetic gas sevofluorane in operating rooms [36, 37].

For surface disinfection at a specific workplace (e.g., treatment or patient rooms), companies often do not have information, such as measurement data, on the exposure to volatile ingredients of the used disinfectants. Estimates of air concentrations based on exposure models are a fast and cost-effective way for exposure assessment of realistic worst case situations instead of commissioning measurements. Knowledge of model quality is required for interpretation of calculated results, however many exposure models have not yet been adequately evaluated and validated.

With the calculations of the different exposure models, we would like to provide guidance on the applicability of exposure models for surface disinfection in healthcare settings and comparable areas. We compared the results of different exposure models (deterministic models: unsteady 1-zone model [38, 39], the ConsExpo model (exposure to vapour model) [27] and the 2-component model [40] as well as the modifying-factor model Stoffenmanager® [22–25]) with data from measurements performed in a specifically described exposure situation. We used different methods in terms of accuracy and conservatism, following examples from the literature [33–36]. For this purpose, we conducted exposure modelling in combination with measurements at one specific workplace.

## Materials and methods

### Experimental setup

In the following, the essential information on the experimental setup in the test chamber, the performance of the surface disinfections, measurements and the analysis of the samples is described (see also for exposure measurements Wegscheider et al. [41]).

#### Description of the test chamber

**Fig. 1:**
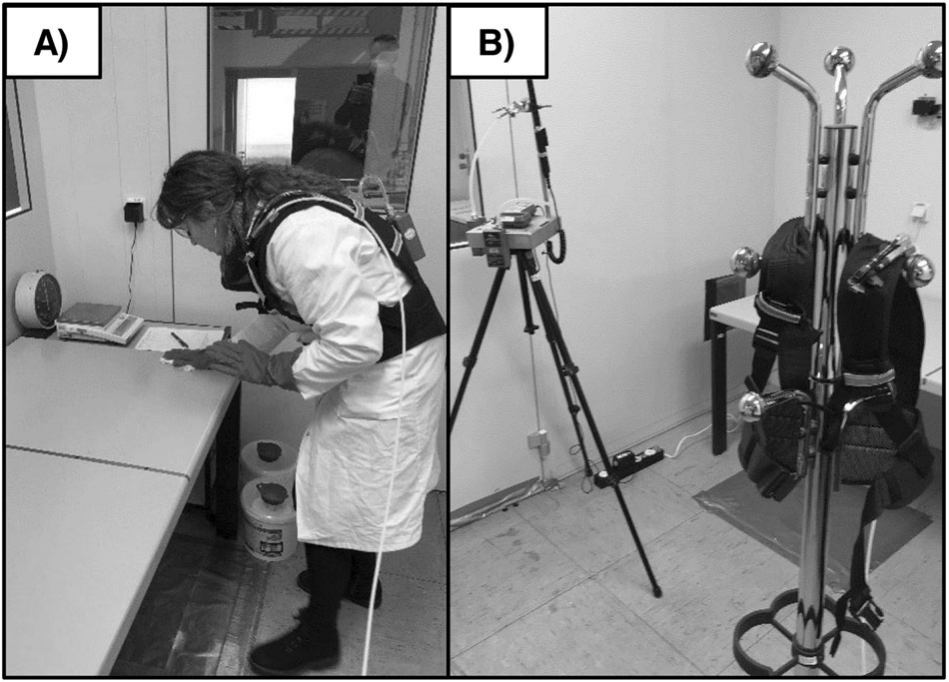
Surface disinfection and measurements. **A** Performance of surface disinfection in the test chamber. **B** Exposure to disinfectant active ingredients was measured using a personal air sampling system (person and clothes rack/ dummy with vest) and stationary air sampling system on the stand. Photos taken by and shown in Wegscheider et al. [41].

The Institute for Occupational Safety and Health (IFA) of the German Social Accident Insurance (DGUV) provided a test chamber used as a room for conducting the surface disinfections with various disinfectants. The room had an area of 13.3 m^2^ (3.8 m × 3.5 m) and a room volume of 39.9 m^3^ (Fig. 1). The natural air exchange rate was determined according to DIN EN ISO 12569 with *λ* = 0.7–0.9/h [42], no additional technical ventilation was used. Between measurements, the exchange of the room air was technically supported with a fan to ensure disinfectant-free air for the next measurement. This was controlled with direct reading instruments and air sampling. The room was equipped with tables on which the disinfectants were applied. The table surfaces were made of standard synthetic materials used for office tables.

#### Description of the surface disinfection

Different surfaces (0.5 m^2^, 2 m^2^, 5 m^2^) were treated with disinfectants based on different active ingredients. For the aldehyde-containing application solution B (No. 3) additionally surfaces of 10 m^2^ and 15 m^2^ were treated, because the measured glutaraldehyde concentrations were below the limit of detection (0.041 mg/m^3^) for almost all measurements in preliminary experiments. The products were provided in different product forms, as wipes in ready-to-use solution or as a concentrate which had to be diluted. The application concentrations are given in Table 1 (details in Supplementary Table 1). The dilution process was not included in the measurements.

**Table 1:** Disinfectants applied for surface disinfection.

No.	Applied disinfectant	Active ingredient	Concentration^a^
**1**	Alcoholic wipes	Ethanol	45 g/100 g
**2**	Aldehyde-containing application solution A	Formaldehyde	0.051 g/100 g
		Glutaraldehyde	0.041 g/100 g
**3**	Aldehyde-containing application solution B	Glutaraldehyde	0.048 g/100 g
**4**	Peroxide wipes	Hydrogen peroxide	4.7 g/100 g
		Peroxyacetic acid	0.15 g/100 g

^a^The concentration of ethanol in the alcoholic wipes was specified by the manufacturer, and the concentration of the active ingredients in the other three applied disinfectants was analysed in the laboratories of the IFA and the German Social Accident Insurance Institution for the foodstuffs and catering industry (BGN).

The concentration describes the concentration of the active ingredients in the ready-to-use solution (wipes) or in the 0.5 wt-% application solution (see Supplementary Table 1 for detailed information).

The amount of disinfectant applied to the surface was determined by weighing the disinfectant-soaked wipes before and after disinfection. The disinfectant was conducted to the surface by hand so that a thin film of liquid was visible on the surface (no puddles). Thus, one disinfection-soaked wipe was used for one square metre of surface, i.e., five wipes for 5 m^2^. One wipe was also used for 0.5 m^2^ (no use of one-half of the wipe, as disinfectant would be lost from the soaked wipe if torn). Each wipe was disposed in a closed waste container in the room immediately after use.

The person performing the surface disinfections wore chemical protection gloves (according to DIN EN ISO 374 [43–46]), safety goggles and a lab coat.

#### Sampling strategy and analytics

The collection of air samples and the subsequent sample analysis were performed in accordance with the validated IFA air sampling methods 7330 for ‘ethanol’, 6045 for ‘aldehydes’ and 8310 for ‘peroxyacetic acid and hydrogen peroxide’ [47–49]. Three repeated measurements were performed for each disinfected surface and for each disinfectant used. Different sampling strategies (personal air sampling and stationary air sampling) were selected for the measurement in the test chamber (Fig. 1). Measurements with personal and stationary air sampling were performed using personal air sampling (PAS) pumps Gilian® LFS 113 DC (*Sensidyne®*^,^, *St. Petersburg/Florida/USA*) with flow rate 0.33 l/min for ethanol or SG 2500 (*GSA Messgerätebau GmbH, Ratingen/Germany*) with flowrate 0.33–1.3 l/min for aldehydes and with flow rate 1.38 l/min for peroxides [47–49]. The PAS pumps were connected via a tube to a sample carrier with different sampling media for ethanol (active carbon type B, *Dräger, Lübeck/Germany*), for aldehydes (Sep-Pak XpoSure, *Waters, Eschborn/Germany*, a 2,4-dinitrophenylhydrazine (DNPH) impregnated absorber) and for peroxides (midget impinger, *SKC, Eighty Four/Pennsylvania/USA*, filled with LC grade water). The sampling time was 15 min, which corresponds to the recommended duration of assessment for short-term exposures in German occupational safety and health regulations [15]. During this time, the surface was disinfected and dried by evaporation. In order to reduce the personal exposure of the person performing the surface disinfection, the person did not remain in the room during the entire sampling period, if the time for disinfection (disinfection time) was shorter than 15 min. In this case, the vest with the personal air sampling system was hung on a clothing rack (as a dummy) in the middle of the room and the person left the room (Fig. 1b). The time to perform disinfection (disinfection time) and the time until the disinfected surface was completely dried (drying time, determined by visual observation and time duration included disinfection time) were stopped with a laboratory timer. Temperature and relative humidity were also determined in the test chamber using the multifunction metre Q-Trak, (*TSI GmbH, Aachen/Germany*) (Supplementary Tables 2 and 3).

The sample carriers were analysed in the laboratories of IFA or of the German Social Accident Insurance Institution for the Foodstuffs and Catering Industry (BGN), respectively. According to method 7330 (ethanol), the activated carbon was extracted with 10 ml of a ternary mixture of dichloromethane, carbon disulfide and methanol (volume fractions 60%, 35%, 5%) for 30 min and filtered through a syringe filter (PTFE, pore size 0.45 µm). Quantitative analysis was performed using internal calibration by gas chromatography (GC) with flame ionisation detection (FID) [47]. According to method 6045 (aldehydes), the DNPH-aldehydes were eluted from the cartridge with acetonitrile and the solution was acidified with phosphoric acid. After a standing time of 48 h, high-pressure liquid chromatography (HPLC) with external calibration and detection via a diode array detector (DAD) was used for quantitative analysis [48]. According to method 8310, the unstable aqueous solution with hydrogen peroxide and peroxyacetic acid was worked up immediately after sampling. Methyl-*p*-tolyl sulfide and a buffer were added to an aliquot of the absorbance solution and allowed to react in the dark for 10 min. Then, triphenylphosphine (TPP) was added to the sample. The corresponding formed oxides (sulfoxide and triphenylphosphine oxide) were analysed by HPLC and DAD and quantitative determination was based on calibration curves with external standard [49].

For each disinfectant, for each disinfected surface and for each sampling strategy used, three measured values (raw data) were received, because three repeated measurements were performed. Thus, the mean of the measured values was calculated (Supplementary Table 3).

### Exposure modelling

The models for the assessment of inhalation exposure were selected according to the following criteria: The parameters of the experimental setup for surface disinfection in the test chamber are very well described, therefore the simple unsteady 1-zone model is suitable. The other exposure models such as ConsExpo, 2-component model and Stoffenmanager® were chosen, because they are well-known online as spreadsheet or web-based application for risk assessment in registration under REACH and BPR as well as in occupational safety and health. As the air exchange rate was determined to be 0.7–0.9/h in the test camber, the lowest rate (0.7/h) was used for calculation to represent the worst case.

#### Unsteady 1-zone model

The modelling of concentration values using the physico-chemical deterministic approach was performed assuming a 1-zone mass-balance-based model under unsteady conditions with homogeneous distribution of the disinfectant active ingredient in the room [38, 39]. The unsteady conditions take into account the short-term inhalation exposure of the person during the performed surface disinfection [38]. The following Eq. (1) was used for the calculation of the mean concentration [39]:

1 $$\bar{{{{{{{\rm{x}}}}}}}_{{{{{{\rm{i}}}}}}}}=\left(\frac{\dot{{{{{{{\rm{m}}}}}}}_{{{{{{\rm{i}}}}}}}}}{{{\dot {{{{{\rm{V}}}}}}}_{{{{{{\rm{air}}}}}}}}}+{{{{{{\rm{x}}}}}}}_{{{{{{\rm{i}}}}}},\,{{{{{\rm{ex}}}}}}}\right)\cdot \left(1-\frac{1-{{{{{{\rm{e}}}}}}}^{-{{{{{\rm{\lambda }}}}}}\cdot ({{{{{{\rm{t}}}}}}}_{1}-{{{{{{\rm{t}}}}}}}_{0})}}{{{{{{\rm{\lambda }}}}}}\cdot ({{{{{{\rm{t}}}}}}}_{1}-{{{{{{\rm{t}}}}}}}_{0})}\right)+\left({{{{{{\rm{x}}}}}}}_{{{{{{\rm{i}}}}}},0}\cdot \frac{1-{{{{{{\rm{e}}}}}}}^{-{{{{{\rm{\lambda }}}}}}\cdot ({{{{{{\rm{t}}}}}}}_{1}-{{{{{{\rm{t}}}}}}}_{0})}}{{{{{{\rm{\lambda }}}}}}\cdot ({{{{{{\rm{t}}}}}}}_{1}-{{{{{{\rm{t}}}}}}}_{0})}\right)$$

The following Eq. (2) was used for the calculation of the current concentration [39]:

2 $${{{{{{\rm{x}}}}}}}_{{{{{{\rm{i}}}}}}}=\left(\frac{\dot{{{{{{{\rm{m}}}}}}}_{{{{{{\rm{i}}}}}}}}}{{{\dot{{{{{\rm{V}}}}}}}_{{{{{{\rm{air}}}}}}}}}+{{{{{{\rm{x}}}}}}}_{{{{{{\rm{i}}}}}},{{{{{\rm{ex}}}}}}}\right)\cdot \left(1-{{{{{{\rm{e}}}}}}}^{-{{{{{\rm{\lambda }}}}}}\cdot ({{{{{{\rm{t}}}}}}}_{1}-{{{{{{\rm{t}}}}}}}_{0})}\right)+\left({{{{{{\rm{x}}}}}}}_{{{{{{\rm{i}}}}}},0}\cdot {{{{{{\rm{e}}}}}}}^{-{{{{{\rm{\lambda }}}}}}\cdot ({{{{{{\rm{t}}}}}}}_{1}-{{{{{{\rm{t}}}}}}}_{0})}\right)$$

With:

$\bar{{{{{{{\rm{x}}}}}}}_{{{{{{\rm{i}}}}}}}}$ = mean concentration of the disinfectant active ingredient in the room air at a certain exposure time

${{{{{{\rm{x}}}}}}}_{{{{{{\rm{i}}}}}}}$ = current concentration of the disinfectant active ingredient in the room air at a certain exposure time

*i* = disinfectant active ingredient

$\dot{{{{{{{\rm{m}}}}}}}_{{{{{{\rm{i}}}}}}}}$ = mass flow [mg/h], i.e., applied amount of disinfectant active ingredient per drying time

${{\dot{{{{{\rm{V}}}}}}}_{{{{{{\rm{air}}}}}}}}$ = air volume flow [m^3^/h], i.e., room volume *V* multiplied by air exchange rate *λ*

*V* = room volume [m^3^]; i.e. 39.9 m^3^

*λ* = air exchange rate [1/h]; i.e. 0.7/h

t_1_−*t*_0_ = time increment [h], i.e. 1 min = 1/60 h

${{{{{{\rm{x}}}}}}}_{{{{{{\rm{i}}}}}},0}$ = current concentration of the disinfectant active ingredient in the air at a certain exposure time that is one time increment shorter than the exposure time for ${{{{{{\rm{x}}}}}}}_{{{{{{\rm{i}}}}}}}$ or ${\bar{{{{{{\rm{x}}}}}}}}_{{{{{{\rm{i}}}}}}}$

${{{{{{\rm{x}}}}}}}_{{{{{{\rm{i}}}}}},{{{{{\rm{ex}}}}}}}$ = current concentration of the disinfectant active ingredient outside the room

The parameters for all disinfectant active ingredients for disinfection of the different surfaces for calculations with the unsteady 1-zone model can be found in Supplementary Tables 4 and 5. The exposure time corresponded to the time for personal air sampling (sampling time). The drying time was shorter than the exposure time in most cases. In these cases, $\dot{{{{{{{\rm{m}}}}}}}_{{{{{{\rm{i}}}}}}}}$ was >0 mg/h during the drying time, but in the period between the end of the drying time to the end of the exposure time $\dot{{{{{{{\rm{m}}}}}}}_{{{{{{\rm{i}}}}}}}}$ was 0 mg/h. For modelling, the mean concentration $\dot{{{{{{{\rm{x}}}}}}}_{{{{{{\rm{i}}}}}}}}$ was determined for the worker’s exposure time of 15 min or for No. 3 by disinfecting 15 m^2^ for an exposure time of 23 min. By summing up the individual concentrations of $\dot{{{{{{{\rm{x}}}}}}}_{{{{{{\rm{i}}}}}}}}$ over the respective time increments, $\dot{{{{{{{\rm{x}}}}}}}_{{{{{{\rm{i}}}}}}}}$ was obtained at the exposure time of 15 min or 23 min. During the exposure time no active ingredient entered the room from outside through doors or windows, that resulted in *x*_i,ex_ = 0.

#### ConsExpo

ConsExpo is a web-based tool originally intended for estimating human exposure to chemicals contained in consumer products. The simulation tool contains various mass-balance-based models for simulating inhalation, dermal and oral exposure. ConsExpo calculations were carried out by the formulas as described in the corresponding manual for ConsExpo’s evaporation model [27]. The formulas were implemented in a spreadsheet editor. Results were cross-checked and match calculations carried out with ConsExpo Web 1.1.1. A tiered approach was chosen. Tier 1 describes rough estimates based on conservative or simplified default values. Tier 2 includes refined assumptions such as calculated mass transfer coefficients and activity coefficients, if available [27]. The possibility to enter the activity coefficient directly into ConsExpo is not existent nor mentioned in its manual. In contrast, the publication of the 2-component model (see below ref. [40]) describes a possibility to introduce activity coefficients by modifying the vapour pressure. This possibility was used for ConsExpo as well to be able to compare the Tier 2 results of ConsExpo and the 2-component model. Consequently, the activity coefficient γ_i_ was used as a factor to modify the vapour pressure of the substance. The corresponding activity coefficients were calculated with XLUNIFAC (ethanol: *γ*_i_ = 7.39 [50]), according to Radl et al. (hydrogen peroxide: *γ*_i_ = 0.30 [51]) or with AIOMFAC (peroxyacetic acid: *γ*_i_ = 2.465 [52–55]). All calculations were carried out for systems containing only water and one substance (ethanol, hydrogen peroxide or peroxyacetic acid).

ConsExpo uses several equations. For the calculation mode ‘Exposure to vapour: evaporation’ two characteristic equations are mentioned here as an example, which describes the evaporation of a substance from a product layer (Eqs. (3) and (4)):

3 $$\frac{{{dA}}_{{air}}}{{dt}}=K\cdot S\cdot \frac{M}{{RT}}\cdot ({p}_{{eq}}-{p}_{{air}})-Q\cdot {V}_{{room}}\cdot {x}_{i}$$

4 $$\frac{{{dA}}_{{prod}}}{{dt}}=-K\cdot S\cdot \frac{M}{{RT}}\cdot ({P}_{{eq}}-{P}_{{air}})+{A}_{{tot}}/{T}_{{app}}\cdot {w}_{f}$$

With:

*A*_air_ = mass of the substance in the air [kg]

*A*_prod_ = mass of the substance in the product [kg]

*t* = time [s]

*K* = mass transfer coefficient of the substance [m/s]

*S* = treated area [m^2^]

*M* = molecular weight [kg/mol]

*R* = gas constant [J/(mol K)]

*T* = temperature [K]

*p*_eq_ = equilibrium vapour pressure [Pa]

*p*_air_ = actual vapour pressure [Pa]

*Q* = room ventilation rate [1/s]

*V*_room_ = room volume [m^3^]

*x*_i_ = concentration of the substance in the air

*A*_tot_ = total amount of used product [kg]

*T*_app_ = application time [s]

*w*_f_ = weight fraction of the substance in the product [fraction]

The results are given as mean event concentration (MEC) by ConsExpo, the mean value of all predicted concentrations over time. Further information can be found in the corresponding RIVM report 2017-0197 [27]. The parameters for Tier 1 and Tier 2 calculations for other disinfectant active ingredients and the different surface areas can be found in Supplementary Tables 6–9.

#### 2-component model

The 2-component model was originally developed in the context of the biocide exposure assessment as a tool running in a spreadsheet editor and published by the Ad hoc Working Group on Human Exposure (HEAdhoc) and is used in context of biocidal product authorisation [56]. It is based on essentially the same equations as those used by the ConsExpo evaporation model, but additionally simulates evaporation of the solvent. This can result in delayed evaporation of the substance of interest, if the substance has a significant lower vapour pressure than its solvent (in this case: water). A more detailed description and discussion of the algorithm can be found in the literature [40]. A tiered approach was used for the 2-component model as well. For Tier 2, the mass transfer coefficient [50] and the activity coefficient (if available) were modified (see above). In addition to the formulas (3 and 4) already given for ConsExpo, two additional formulas are added to describe the behaviour of the solvent in the 2-component model (Eqs. (5) and (6)) [56]:

5 $$\frac{{{dm}}_{{air},\,{solv}.}}{{dt}}\,=\,	 K\cdot S\cdot \frac{{M}_{{solv}.}}{{RT}}\cdot ({P}_{{eq},\,{solv}.}-{P}_{{air},\,{solv}.})\\ 	 -Q\cdot {V}_{{room}}\cdot {x}_{{air},\,{solv}.}+Q\cdot {V}_{{room}}\cdot {x}_{{fresh\; air},\,{solv}.}$$

6 $$\frac{{{dm}}_{{prod},\,{solv}.}}{{dt}}\,=\,-K\cdot S\cdot \frac{{M}_{{solv}.}}{{RT}}\cdot ({P}_{{eq},\,{solv}.}-{P}_{{air},\,{solv}.})+{A}_{{tot}}/{t}_{{app}}\cdot (1-{w}_{f})$$

With:

*m*_air, solv._ = amount (mass) of solvent in the air [kg]

p_eq, solv._ = equilibrium vapour pressure of solvent in the liquid product mixture [Pa = kg/(m s^2^)]

*p*_air, solv._ = vapour pressure of solvent in the air [Pa]

*x*_air, solv._ = concentration of the solvent in the air

*x*_fresh air, solv._ = humidity of the fresh air exchanging the air in the room [kg/m^3^]

*m*_prod, solv._ = amount (mass) of solvent in the air [kg]

*t*_app_ = application time (s)

*M*_solv._ = molecular mass of the solvent [kg/mol]

The results are given as MEC by the 2-component model, the mean value of all predicted concentrations over time. The parameters for Tier 1 and Tier 2 calculations for the other disinfectant active ingredients and different surface areas can be found in Supplementary Tables 6–9.

#### Stoffenmanager®

The Stoffenmanager® exposure model is based on the source-receptor approach proposed by Cherrie and Schneider [19]. A multiplier is given for the exposure-determining factors such as emission potential of the used products and the performed activity, room size and ventilation or applied local control measures [22]. Equation (7) is used to calculate the total concentration score *C*_t_.

7 $${{{{{{\rm{C}}}}}}}_{{{{{{\rm{t}}}}}}}=\left[\left({{{{{\rm{E}}}}}}\cdot {{{{{\rm{a}}}}}}\right)+\left({{{{{\rm{E}}}}}}\cdot {{{{{\rm{H}}}}}}\cdot {{{{{{\rm{\eta }}}}}}}_{{{{{{\rm{lc}}}}}}}\cdot {{{{{{\rm{\eta }}}}}}}_{{{{{{\rm{gv}}}}}}\_{{{{{\rm{nf}}}}}}}\right)+\left({{{{{\rm{E}}}}}}\cdot {{{{{\rm{H}}}}}}\cdot {{{{{{\rm{\eta }}}}}}}_{{{{{{\rm{lc}}}}}}}\cdot {{{{{{\rm{\eta }}}}}}}_{{{{{{\rm{gv}}}}}}\_{{{{{\rm{ff}}}}}}}\right)\right]\cdot {{{{{{\rm{\eta }}}}}}}_{{{{{{\rm{imm}}}}}}}$$

With:

*C*_t_ = total concentration score

*E* = intrinsic emission score of the product

*a* = multiplier for the relative influence of background sources

*H* = handling (or task) score

*η*_lc_ = multiplier for the effect of local control measures on the exposure

*η*_gv_nf_ = multiplier for the effect of general ventilation in relation to the room size on the exposure to near-field sources

*η*_gv_ff_ = multiplier for the effect of general ventilation in relation to the room size on the exposure to far-field sources

*η*_imm_ = multiplier for the reduction of exposure due to control measures at the worker

The estimation of exposure in mg/m³ is done by applying a regression formula between the calculated scores and measurement values. Initially, about 700 measurements were used to derive the quantitative algorithms [23]. Later, a cross-validation and further refinement of the model based on almost 1000 measurements was performed [25].

To describe the surface disinfection with alcoholic wipes in the test chamber, the selected input parameters for Stoffenmanager® are listed in Supplementary Table 10.

### Methods to assess the quality of exposure models

For assessing the quality of the simulated results, two different methods were applied in context with earlier works in this research field. On the one hand, the direct comparison of simulated value versus measured value was conducted [34]. On the other hand, the level of conservatism of the applied models was assessed [33].

#### Comparison of modelled to measured data (accuracy)

To evaluate the different exposure models, a comparison (PRED/EXP) of the modelled value (PRED = predicted value) was made with the mean of the measured values from personal air sampling (EXP) [34]. By comparing modelled to measured values, the overestimation or the underestimation can be quantified. When PRED is identical with EXP, the result is 1. In case of underestimation PRED/EXP is <1 and in case of overestimation PRED/EXP is > 1. For surface disinfection for which no mean of the measured values could be determined (all results below the detection limit), no PRED/EXP could be conducted. The highest model accuracy is given by PRED/EXP = 1 (accurate). The exposure model shows a good accuracy with 0.5 < PRED/EXP < 5 and an acceptable accuracy with 0.1 < PRED/EXP < 10. The ranges are chosen based on proposed criteria from the literature [34], which are also recommended in Germany for assessment of inhalation exposure [57]. PRED/EXP of maximum of 5 represents a recommend overestimation in occupational exposure assessment [34, 57].

#### Level of conservatism

The level of conservatism of an exposure estimation tool is another way of determining the quality of exposure models [33]. The measured values (raw data) from personal and stationary air sampling (EXP) were plotted against each modelled value of the deterministic models. The 1:1 line represents agreement between the modelled value to the measured value. The data points above the 1:1 line describe an underestimation of the modelled value to the measured values (PRED < EXP). If the data points are below the 1:1 line, the exposure model overestimates the measured values (PRED > EXP). For surface sizes, for which (almost) all measured value of a disinfectant active ingredient (e.g., glutaraldehyde No. 2) were below the detection limit, no scatter plots were created. Conservatism of a model can be defined as high (≤10% of measured values exceed the model estimate), medium (11% ≤ 25%) or low (>25%) [33].

## Results

**Fig. 2:**
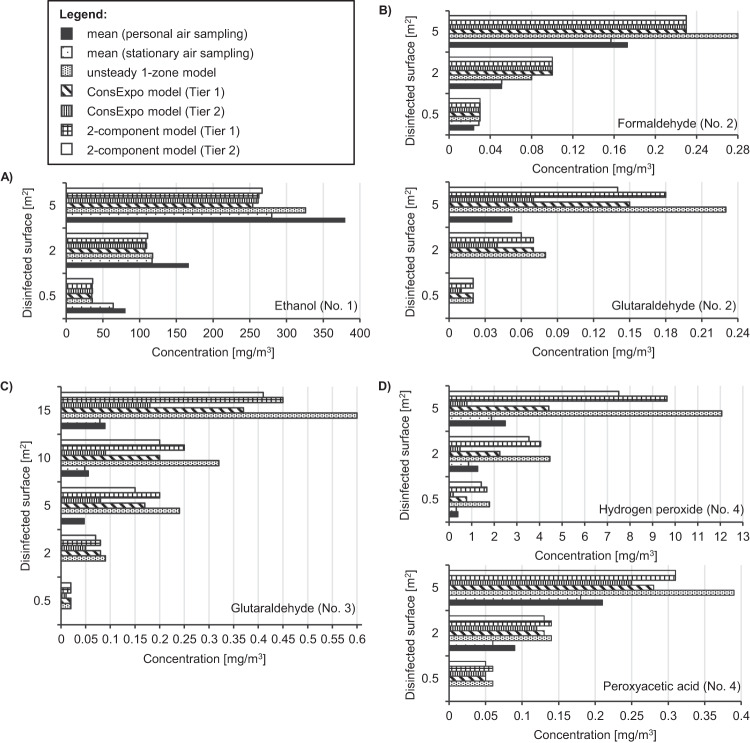
Visualised measured and modelled values of surface disinfection. Visualisation of mean values of measured data from personal and stationary air sampling and modelled values of the unsteady 1-zone model, the ConsExpo model and the 2-component model for (**A**) ethanol (No. 1), **B** formaldehyde and glutaraldehyde (No. 2), **C** glutaraldehyde (No. 3) and **D** hydrogen peroxide and peroxyacetic acid (No 4).

Table 2 shows the mean of the measured values of personal and stationary air sampling as well as the modelled values of the deterministic models (for details see Supplementary Table 2–9). These results are also visualised in Fig. 2. During measurement performance the temperature in the test chamber varied in range between 19.2 °C and 21.7 °C (292.35–294.85 K) and the relative humidity between 26.7% to 55%.

**Table 2:** Mean values of measured data from personal and stationary air sampling and modelled values with the unsteady 1-zone model, the ConsExpo model and the 2-component model as well as the comparison of the modelled values (PRED) with the mean of the measured values from personal air sampling (EXP).

No.	Disinfectant active ingredients	Disinfected surface [m^2^]	Mean value (EXP) [mg/m^3^]		Modelled values (PRED) [mg/m^3^]					Comparison (PRED/EXP_personal air sampling_)^a^				
			Personal air sampling	Personal air sampling	Unsteady 1-zone model	ConsExpo (Tier 1)	ConsExpo (Tier 2)	2-component model (Tier 1)	2-component model (Tier 2)	Unsteady 1-zone model	ConsExpo (Tier 1)	ConsExpo (Tier 2)	2-component model (Tier 1)	2-component model (Tier 2)
**1**	Ethanol	0.5	81	64	35.53	34.92	35.87	35.21	35.99	0.44	0.43	0.44	0.43	0.44
		2	167	117	117.67	107.17	109.52	108.73	110.94	0.7	0.64	0.66	0.65	0.66
		5	380	280	325.76	254.01	261.28	262.32	266.9	0.86	0.67	0.69	0.69	0.7
**2**	Formaldehyde	0.5	0.024	0.029	0.03	0.03	0.03	0.03	0.03	1.28	1.27	1.27	1.27	1.27
		2	0.051	0.051	0.08	0.1	0.1	0.1	0.1	1.55	1.9	1.9	1.9	1.9
		5	0.173	0.157	0.28	0.23	0.23	0.23	0.23	1.64	1.31	1.31	1.31	1.31
	Glutaraldehyde	0.5	n. d.	n. d.	0.02	0.02	0.01	0.02	0.02	-	-	-	-	-
		2	n. d.	n. d.	0.08	0.07	0.04	0.07	0.06	-	-	-	-	-
		5	0.052	n. d.	0.23	0.15	0.07	0.18	0.14	4.38	2.94	1.41	3.38	2.74
**3**	Glutaraldehyde	0.5	n. d.	n. d.	0.02	0.02	0.01	0.02	0.02	-	-	-	-	-
		2	n. d.	n. d.	0.09	0.08	0.05	0.08	0.07	-	-	-	-	-
		5	0.047	n. d.	0.24	0.17	0.08	0.2	0.15	5.09	3.67	1.8	4.15	3.24
		10	0.055	0.048	0.32	0.2	0.09	0.25	0.2	5.81	3.69	1.55	4.58	3.6
		15	0.089	0.079	0.6	0.37	0.18	0.45	0.41	6.69	4.74	2.32	5.74	5.25
**4**	Hydrogen peroxide	0.5	0.39	0.32	1.79	0.77	0.18	1.67	1.43	4.59	1.99	0.46	4.27	3.67
		2	1.26	0.86	4.46	2.25	0.49	4.05	3.53	3.53	1.79	0.39	3.21	2.8
		5	2.48	1.88	12.06	4.41	0.79	9.64	7.5	4.86	1.78	0.32	3.89	3.02
	Peroxyacetic acid	0.5	n. d.	n. d.	0.06	0.05	0.05	0.06	0.05	-	-	-	-	-
		2	0.09	0.06	0.14	0.13	0.12	0.14	0.13	1.58	1.4	1.32	1.5	1.46
		5	0.21	0.18	0.38	0.28	0.25	0.31	0.31	1.83	1.32	1.2	1.5	1.45

*n.d.* Not determinable, i.e., for the concentration is below the analytical detection limit (glutaraldehyde: 0.041 mg/m^3^, peroxyacetic acid: 0.05 mg/m^3^.

^a^In the case, that the mean value of the personal air sampling was not determinable (n.d.), no PRED/EXP could be determined.

In general, the mean of the measured values was higher in the case of personal air sampling than in the case of stationary air sampling. This effect was particularly observed for ethanol. The modelled values of the unsteady 1-zone model for ethanol were lower than the mean values, except for the mean values of the stationary air sampling after disinfecting 2 m^2^ or 5 m^2^. For all calculations using the Tier 1 or Tier 2 approach of the ConsExpo model or the 2-component model, an underestimation of the measured values for ethanol was observed. For the other disinfectants, the modelled values of the active ingredients were above the mean values of personal or stationary air sampling, except for the ConsExpo Tier 2 calculation of hydrogen peroxide.

In order to make quantitative statements about the accuracy of the exposure models, PRED/EXP was determined (Table 2). All deterministic models showed a PRED/EXP of minimum 1.2 and maximum 1.9 for formaldehyde and peroxyacetic acid. For the ConsExpo Tier 2 calculation of glutaraldehyde PRED/EXP was <2.32, but for the other deterministic models PRED/EXP was between 2.74 and 6.69. Similarly, for hydrogen peroxide, the ConsExpo Tier 2 calculation resulted in PRED/EXP <0.5, but for the other models between 1.78 and 4.86. In the case of ethanol, PRED/EXP for each deterministic model when compared to the mean value of personal air sampling was 0.43 or 0.44 for disinfection of 0.5 m^2^ and between 0.64 to 0.86 for disinfection of 2 m^2^ and 5 m^2^. The underestimation of the modelled values for ethanol was smaller when these values were compared with the mean value of stationary air sampling (Supplementary Table 11). PRED/EXP were 0.55 or 0.56 for 0.5 m^2^ and for 2 m^2^ and 5 m^2^ between 0.92 and 0.95 for ConsExpo and 2-component model as well as for the unsteady 1-zone model 1.00 for 2 m^2^ and 1.16 for 5 m^2^. As example for the unsteady 1-zone model, PRED/EXP was calculated with the modelled values under additionally ventilation conditions (*λ* = 0.8/h and 0.9/h). In general, the insignificant better ventilation results in slightly smaller PRED/EXP (Supplementary Table 12).

Since Stoffenmanager® uses the same activity ‘Handling of liquids where only small amounts of product may be released’ for all sizes of the disinfected surface, only one air concentration could be calculated. The exposure level (90th percentile = 726 mg/m^3^) for ethanol was in the same order of magnitude than the measured values. Within Stoffenmanager® it is possible to obtain not only one modelled value, but an estimate for the whole exposure distribution (Table 3, Supplementary Fig. 1). The exposure distribution for ethanol was also used to determine PRED/EXP (Table 3). The 50th percentile of 0.5 m^2^ was almost identical to the measured value (PRED/EXP = 0.99). PRED/EXP was between 0.68 to 3.17 (from 5 m^2^ to 0.5 m^2^) for the 75th percentile, 4.35 (2 m^2^) and 1.91 (5 m^2^) for the 90th percentile and lastly 3.58 (5 m^2^) for the 95th percentile. For small 50th percentile and surface size with ≥2 m^2^ PRED/EXP was <0.5 and opposite for large percentiles (90th and 95th) and surface size with ≤2 m^2^ PRED/EXP was >8.

**Table 3:** Modelled exposure distribution by Stoffenmanager® for ethanol (No. 1) and the comparison with the mean value from personal air sampling (EXP) for the different percentiles and surface sizes.

Percentile	Modelled values [mg/m^3^]	Comparison (PRED/EXP_personal air sampling_)		
		0.5 m^2^	2 m^2^	5 m^2^
50th	80.42	0.99	0.48	0.21
75th	257	3.17	1.54	0.68
90th	726	8.96	4.35	1.91
95th	1360	16.79	8.14	3.58

**Fig. 3:**
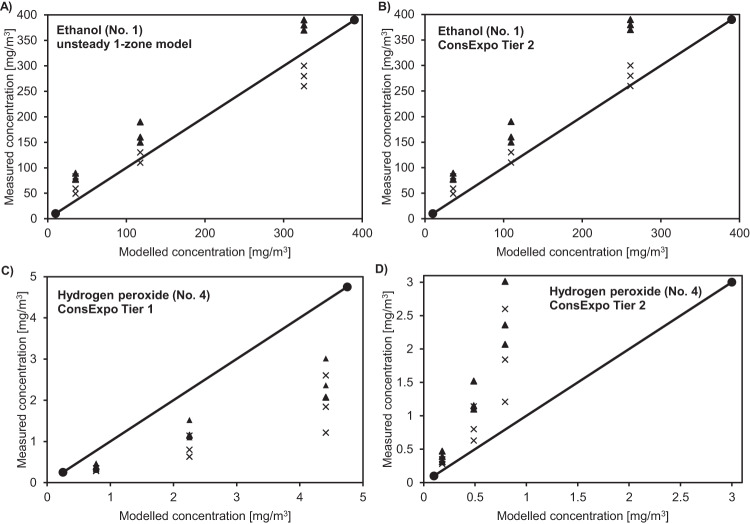
Selected scatter plots of measured to modelled data. Measured data from ethanol (No. 1) of personal air sampling (▲) and stationary air sampling (**x**) plotted against the modelled data of (**A**) the unsteady 1-zone model and **B** ConsExpo (Tier 2 calculation) as well as measured data from hydrogen peroxide (No. 4) of personal air sampling (▲) and stationary air sampling (**x**) plotted against the modelled data of (**C**) ConsExpo (Tier 1 calculation) and **D** ConsExpo (Tier 2 calculation), shown as selected examples. Each scatter plot includes data for the three different sizes of the disinfected surface.

Furthermore, scatter plots (selected examples in Fig. 3 and complete overview in Supplementary Figs. 2–6) were created with the raw measurement data and the modelled values of the deterministic models to visualise the level of conservatisms of the exposure models. At first, the scatter plots for each of the three independently performed measurements (personal as well as stationary air sampling) for a disinfectant and a disinfected surface size illustrate the deviations between the type of air sampling on the one hand and the independent replicates on the other hand. All deterministic models except ConsExpo (Tier 2 calculation) for hydrogen peroxide showed that the modelled values for the disinfectant active ingredients glutaraldehyde, hydrogen peroxide and peroxyacetic acid overestimated the measured values. None of the measured values exceeded the model estimates, however, all measured values (100%) exceeded the estimates of the ConsExpo model (Tier 2 calculation) for hydrogen peroxide. Additionally, almost all measured values laid above the modelled values for ethanol (unsteady 1-zone 72%, ConsExpo Tier 1 100% and Tier 2 94%, 2-component model Tier 1 94% and Tier 2 83%) and 12% of measured values of formaldehyde exceeded the estimates of all models.

## Discussion

The conditions of the surface disinfection such as the performance, the working environment, the ventilation situation as well as the measurements carried out were documented in detail (see section A) ‘Experimental Set Up of Materials and Methods’ as well as Wegscheider et al. [41]). These exact parameters of the specific workplace formed the starting point for the calculation of the concentrations with different exposure models and to assess the quality of the models.

For the measurement data, it was to be expected that the mean values were higher in the case of personal air sampling than in the case of stationary air sampling, since the stationary sampling system was further away from the disinfected surface than the person performing the disinfection. The mean values of the personal air sampling were chosen for comparison, because these are more relevant with regards to occupational safety and health, although the deterministic models used here assume a homogeneous distribution of the substance in the room.

The comparisons made showed differences between the measured values and the modelled values - especially overestimates or, in the case of ethanol, underestimates—which are usually to be expected and explained. Estimating the evaporation of disinfectants—or volatile chemicals in general—from a surface into indoor air using exposure models can be challenging. When using exposure models, it should be kept in mind that any exposure model is based on assumptions and has limitations in some cases [21].

One way to look more closely at the exposure model quality is to describe how accurately the modelled values compare to the measured ones. From an occupational safety and health perspective, underestimation of exposure by modelling should be avoided because it means potential underestimation of workplace hazards. Certain degree of overestimation like fivefold is usually considered acceptable, if not desirable to address uncertainties associated with the models. However, if an overestimation is too pronounced, it might result in more strict protective measures which may pose a burden for employees. Taking into account the standards defined so far by Spinazzè et al. [34], the maximum overestimation of factor 10 was not reached in the described exposure modelling by the three deterministic models or Stoffenmanager®. The overestimations of the modelled values were mostly below the recommended factor of 5 [34] and thus, the models considered are suitable for exposure assessment in surface disinfection. For the active ingredients formaldehyde, glutaraldehyde, hydrogen peroxide and peroxyacetic acid, the deterministic models overestimated from good to acceptable degrees. In general, a good to acceptable accuracy could be observed between the estimates of the different models and the measured values for the one specific considered exposure situation of surface disinfection. This shows an advantage of a comparative project with measurements and exposure modelling, as many parameters were recorded and therefore could be entered into the exposure models.

In the case of ethanol, the deterministic models underestimated the exposure. It can be assumed that ethanol evaporates rapidly into the breathing zone of the worker, but is not yet completely homogeneously distributed in the room within the short exposure time. This is supported by comparison of the values of the personal air sampling with the mean of the measured values of the stationary air sampling. This shows that the concentration of ethanol is very inhomogeneous within the room. If the measured values of the stationary air sampling would be used to calculate PRED/EXP, the results would fit a better to the measured values in terms of less underestimation. As this work is written in context of occupational safety and health with a focus on protecting workers, we are not pursuing this path any further. Instead the focus remains on the measured values of the personal air sampling. As a consequence, the applicability of the described deterministic models for an adequate exposure assessment must be viewed critically for the disinfection of smaller surfaces with ethanol.

The mass flow, i.e., the amount of disinfectant released into the indoor air, could be accurately calculated with some deterministic models such as the unsteady 1-zone model mentioned here. A potential source for overestimation of the exposure models are chemical reactions of the substances, for which the evaporation should be simulated. A typical example would be peroxide compounds, such as hydrogen peroxide or peroxyacetic acid. If a substance reacts to a certain extent on the surface (e.g. absorption) or in the air (e.g. decay), this will cause the models to overestimate more, since the chemical reaction was not taken into account in the modelling.

The unsteady 1-zone model determines the mass transfer from surface into air by using a fixed evaporation time and a corresponding amount of substance on the surface. This results in a fixed evaporation rate. ConsExpo and the 2-component model consider mass transfer between surface and (indoor) air by using a mass transfer coefficient as a parameter. In ConsExpo the generic default value is 10 m/h. For the Tier 2 calculations, mass transfer coefficients were determined according to the method of Sparks as harmonised within ECHA´s HEAdhoc recommendation 6 [58]. As the context of this work is occupational safety and health, we followed the recommendation and did not evaluate other methods for the determination of the mass transfer coefficient and their impact on the simulation results.

In addition to the mass transfer coefficient, the activity coefficient could also be modified (Tier 2 approach of ConsExpo and 2-component model), which may lead to delayed evaporation and thus underestimation for not highly volatile substances such as hydrogen peroxide. It should be stressed that the consideration of the activity coefficient in Tier 2, introduced by modification of the vapour pressure, is not originally foreseen or recommended by the ConsExpo manual.

Further, the air exchange rate selected for modelling must be considered for comparison with measured values. The choice of the lower limit of an air exchange rate (e.g. 0.7/h) represents the worst case of ventilation conditions relevant in occupational safety and health. A significant overestimation of the modelled values can be excluded if the upper limit of the air exchange rate (0.9/h) is close to the lower limit.

In contrast to the previously discussed deterministic models, Stoffenmanager® is an example of a modifying-factor model. Stoffenmanager® estimated the whole exposure distribution for ethanol, which allowed a more detailed interpretation. The smaller the percentile of the Stoffenmanager® exposure distribution, the better the quality of these estimations compared to the measured values for disinfection of ≤2 m^2^. Higher percentiles were more comparable with the measured values for lager surfaces (≥2 m^2^) and thus, showed a good accuracy. Therefore, the Stoffenmanager® exposure model is suitable for the assessment of the inhalation exposure of ethanolic disinfectants during surface disinfection for this particular experimental setup. However, the Stoffenmanager® exposure model is not applicable for very reactive substances as peroxides or for highly diluted aldehydes, since the partial vapour pressure in the application solution differs highly from the vapour pressure of the pure substances. For ingredients in very dilute application solutions like aldehydes, the exposure modelling with Stoffenmanager® would probably also be possible if the specific partial vapour pressures are known (e.g. calculation using Henry’s law). However, it should be noted that the exposure model in Stoffenmanager® was calibrated for substances where the vapour pressure of a diluted solution is approximately the same as the vapour pressure of the pure substance. To date, there is no comprehensive validation study available showing that Stoffenmanager® is applicable to substances where the vapour pressure in high dilution differs significantly form the pure substance.

Besides Stoffenmanager®, ART (Advanced REACH Tool) is another modifying-factor model for the exposure assessment, which is also recommended for accessing inhalation exposures for certain scenarios in the regulatory context [26, 59]. However, ART is optimised to describe the exposure during a 480-min shift with continued application [26] and not for describing a short-term surface disinfection. Additionally, certain important information about our specific workplace scenario (small surface, small substance amount, single application) cannot not be entered into the user interface. Initial trials, carried out despite these considerations, result in a large overestimation of the air concentration. Therefore, ART was not further investigated for surface disinfection in this study.

The conservatism of an exposure model and the description of its levels can also be used to consider the quality of the model [33]. All deterministic models showed in general high conservatism for exposure to glutaraldehyde, peroxyacetic acid and hydrogen peroxide and medium conservatism for exposure to formaldehyde. Low conservatism was present in all deterministic models for exposure to ethanol and in ConsExpo Tier 2 calculation for hydrogen peroxide, with almost all measured values exceeding the modelled ones. In contrast to previous assessments of model conservatism in other studies [30], only one specific scenario was considered, meaning that conservatism was not determined using extensive measured data. Despite this small data set, we determined conservatism for the deterministic models, but it could only be discussed in a limited way. We did not consider conservatism for Stoffenmanager®, because the model uses the same activity for all three sizes of the disinfected surface and thus, only one modelled value could be calculated for ethanol. In particular, we used the scatter plots to show the small deviations of the measured data from personal and stationary air sampling to the modelled values. As well as to show the small differences in exposure of a worker when the actually identical surface disinfection is performed three times independently of each other. These exposure variabilities must also be considered for the assessment of inhalation exposure and the derivation of sufficient protective measures in occupational safety and health.

## Conclusion

In order to investigate the quality of estimates of different exposure models used for the assessment of inhalation exposure resulting from surface disinfection, the results of several exposure models (three deterministic models [27, 39, 40] and Stoffenmanager® [22–25]) were compared in terms of accuracy and conservatism with measured values. This approach was conducted for the specific exposure scenario of surface disinfection in healthcare or similar settings.

In general, the three deterministic models (unsteady 1-zone model, ConsExpo Tier 1/Tier 2 and the 2-component model Tier 1/Tier 2) appear to be suitable to predict the air concentration of disinfectant active ingredients formaldehyde, glutaraldehyde, hydrogen peroxide and peroxyacetic acid, used for surface disinfection of small areas. The three models overestimated the inhalation exposure of the aldehydes and the peroxides, thus in terms of occupational safety and health, sufficient protective measures can be derived. The cases where the modelled values have been underestimated by the simulation tools should alert the reader to be cautious, as this means that inhalation hazard for workers may be overlooked. The results obtained for hydrogen peroxide in the ConsExpo Tier 2 calculation indicate that using ConsExpo with vapour pressures refined to account for activity coefficients, as well as with refined mass transfer coefficients, should be avoided. Furthermore, the three deterministic models are not suitable for assessing the inhalation exposure of ethanol because the models underestimated the air concentrations recorded with personal air sampling and thus also, the inhalation hazard in the breathing zone of a worker. Since, ethanol is a well-known active ingredient for disinfection of small surfaces and is used extensively in health services, a suitable exposure model is needed. For this, Stoffenmanager® showed good to acceptable results for assessing inhalation exposure during surface disinfection with ethanol.

The combined planning of measurement and modelling proved to be advantageous, as the parameters necessary for the different models were recorded in detail. Further studies of this kind may help to substantiate the applicability of exposure models to assess occupational inhalation exposure not only during disinfection but also during work with other volatile hazardous substances.

### Supplementary information

Supplementary Information

## Data Availability

All data generated or analysed during this study are included in this published article and its supplementary information file.
