# Supplementary material for: Occupational inhalation exposure during surface disinfection—exposure assessment based on exposure models compared with measurement data
Source: J Expo Sci Environ Epidemiol. 2023 Dec 25;34(2):345–55. doi: 10.1038/s41370-023-00633-y (PMC11142908; doi:10.1038/s41370-023-00633-y)
Supplement: Supplementary file 1 — Supplementary Information [file 41370_2023_633_MOESM1_ESM.pdf]

Supplementary Information

Occupational inhalation exposure during surface disinfection – exposure assessment based on exposure models compared with measurement data

Lea Anhäuser[1], Benedikt Piorr[2], Mario Arnone[3], Wolfgang Wegscheider[1], Johannes Gerding[1]

[1] German Social Accident Insurance Institution for the Health and Welfare Services (BGW), Department for Occupational Medicine, Hazardous Substances and Public Health, Pappelallee 33/35/37, 22089 Hamburg, Germany

[2] Federal Institute for Occupational Safety and Health (BAuA), Unit Exposure Assessment Biocides, Friedrich-Henkel-Weg 1-25, 44149 Dortmund, Germany

[3] Institute for Occupational Safety and Health (IFA) of the German Social Accident Insurance (DGUV), Section Exposure Monitoring-MGU, Alte Heerstrasse 111, 53757 Sankt Augustin, Germany

Supplementary Table 1: Applied disinfectants for surface disinfection.

No.	Applied disinfectant	Product name	Manufacturer	Product form provided by the manufacturer	Application form	Active ingredient	Concentration in application solution**
1	Alcoholic wipes	Descosept pur wipes RTU	Dr. Schuhmacher GmbH (Malsfeld/Germany)	Wipes in ready-to-use solution	Similar to product form	Ethanol	45 g / 100 g
2	Aldehyde-containing concentrate A	Aldasan	Lysoform Dr. Hans Rosemann GmbH (Berlin/Germany)	Concentrate	Concentrate diluted to 0.5 wt-% application solution*	Formaldehyde	0.051 g / 100 g
						Glutaraldehyde	0.041 g / 100 g
3	Aldehyde-containing concentrate B	Incidin rapid	Ecolab Deutschland GmbH (Monheim am Rhein/Germany)	Concentrate	Concentrate diluted to 0.5 wt-% application solution*	Glutaraldehyde	0.048 g / 100 g
4	Peroxide wipes	Mikrozid® PAA wipes	Schülke & Mayr GmbH (Norderstedt/Germany)	Wipes in ready-to-use application solution	Similar to product form	Hydrogen peroxide	4.7 g / 100 g
						Peroxyacetic acid	0.15 g / 100 g

* A wipe dispenser system, which means a bucket with a wipe roll (Hygoclean, Franz Mensch GmbH, Buchloe/Germany) was used for disinfecting the surfaces with the diluted 0.5 wt-% application solution.

** The concentration of ethanol in the alcoholic wipes was specified by the manufacturer, the concentration of the active ingredients in the three other applied disinfectants was determined in the laboratories of the IFA and the BGN.

Supplementary Table 2: Mean values of measured parameters during measurement of different active ingredients in different disinfectants during surface disinfection. Each mean value of a parameter is composed of three measurements.

**No.**	**Disinfectant active ingredient**	**Disinfected surface [m²]**	**Temperature [°C] (in Kelvin)**	**Relative humidity [%]**	**Amount of disinfectant applied [g]**	**Disinfection time [min]***	**Drying time [min]****
**1**	Ethanol	0.5	21.4 (294.55)	37.8	3.56	0.4	8.0
		2	21.5 (294.65)	43.4	11.7	2.5	10
		5	21.2 (294.35)	55	32.5	6.5	15
**2**	Formaldehyde	0.5	21.2 (294.35)	45.4	2.63	0.4	13
		2	21.2 (294.35)	35.5	8.96	2.5	10
		5	21.5 (294.65)	32.7	24.3	6.5	13
	Glutaraldehyde	0.5	21.2 (294.35)	45.4	2.63	0.4	13
		2	21.2 (294.35)	35.5	8.96	2.5	10
		5	21.5 (294.65)	32.7	24.3	6.5	13
**3**	Glutaraldehyde	0.5	21.5 (294.65)	34.2	2.28	0.4	7.0
		2	21.7 (294.85)	35.5	8.48	2.5	12
		5	20.7 (294.85)	44.2	23.2	6.5	15
		10	21.6 (294.75)	41.7	45.5	15	22
		15	20.8 (293.95)	26.7	77.0	22	30
**4**	Hydrogen peroxide	0.5	19.2 (292.35)	37.5	1.78	0.4	2.0
		2	19.8 (292.95)	33.4	4.35	2.5	5.0
		5	20.1 (293.25)	31.3	11.6	6.5	8.0
	Peroxyacetic acid	0.5	19.2 (292.35)	37.5	1.78	0.4	2.0
		2	19.8 (292.95)	33.4	4.35	2.5	5.0
		5	20.1 (293.25)	31.3	11.6	6.5	8.0

*Including set-up time with removal, weighing and disposal of wipes.
** Determined by visual observation, includes disinfection time.

Supplementary Table 3: Raw measurement data for the disinfectant active ingredients of the performed surface disinfection. The measurement for one chosen active ingredient and one chosen disinfected surface was repeated three times. Further, the mean of the measured values was calculated.

**No.**	**Disinfectant active ingredient**	**Disinfected surface [m²]**	**Sampling time [min]**	**Raw data of personal air sampling [mg/m³]**				**Raw data of stationary air sampling [mg/m³]**			
				**1**	**2**	**3**	**Mean**	**1**	**2**	**3**	**Mean**
**1**	Ethanol	0.5	15	78	77	89	**81**	49	83	59	**64**
		2	15	150	190	160	**167**	110	130	110	**117**
		5	15	380	370	390	**380**	260	280	300	**280**
**2**	Formaldehyde	0.5	15	0.028	<0.021	0.022	**0.024**	0.033	0.022	0.031	**0,029**
		2	15	0.043	0.044	0.065	**0.051**	0.053	0.049	0.050	**0.051**
		5	15	0.190	0.140	0.190	**0.173**	0.150	0.120	0.200	**0.157**
	Glutaraldehyde	0.5	15	<0.041	<0.041	<0.041	**n. d.**	<0.041	<0.041	<0.041	**n. d.**
		2	15	<0.041	<0.041	<0.041	**n. d.**	<0.041	<0.041	<0.041	**n. d.**
		5	15	0.052	<0.041	0.051	**0.052**	<0.041	<0.041	<0.041	**n. d.**
**3**	Glutaraldehyde	0.5	15	<0.041	<0.041	<0.041	**n. d.**	<0.041	<0.041	<0.041	**n. d.**
		2	15	<0.041	<0.041	<0.041	**n. d.**	<0.041	<0.041	<0.041	**n. d.**
		5	15	0.047	<0.041	<0.041	**0.047**	<0.038	<0.041	<0.041	**n. d.**
		10	15	0.056	0.058	0.052	**0.055**	<0.041	0.053	0.043	**0.048**
		15	23*	0.080	0.090	0.096	**0.089**	0.071	0.078	0.088	**0.079**
**4**	Hydrogen peroxide	0.5	15	0.39	0.31	0.47	**0.39**	0.28	0.34	0.35	**0.32**
		2	15	1.15	1.52	1.10	**1.26**	0.63	1.14	0.80	**0.86**
		5	15	2.07	3.01	2.36	**2.48**	1.21	2.60	1.84	**1.88**
	Peroxyacetic acid	0.5	15	<0.05	<0.05	<0.05	**n. d.**	<0.05	<0.05	n.d.	**n. d.**
		2	15	0.10	0.082	0.087	**0.09**	<0.05	0.069	0.061	**0.06**
		5	15	0.20	0.24	0.18	**0.21**	0.16	0.22	0.16	**0.18**

n. d. = Not determinable, i.e., for glutaraldehyde the concentration is below the analytical detection limit of 0.041 mg/m³ and for peroxyacetic acid below 0.05 mg/m³.
*For the disinfection of the area of 15 m², the sampling time was increased to 23 min, because the person needed longer for the surface disinfection.

Supplementary Table 4: Unsteady 1-zone model parameters used for calculations and corresponding results for disinfectants No. 1 to No. 3.

**Disinfected surface [m²]**	**Product amount [g]**	**Weight fraction [%]**	**Drying time [min]**	**Exposure time (=Sampling time) [min]**	**Mass flow during drying time [mg/h]**	**Mass flow between end of drying time to end of exposure time [mg/h]**	**Room volume [m³]**	**Air exchange rate [1/h]**	**Time increment [h]**	**$x_{i,0}$ for one time increment shorter than exposure time [mg/m³]**	**$\bar{x}_i$ for exposure time [mg/m³]**
**No. 1 – Ethanol**											
0.5	3.56	45	8	15	12015	0	39.9	0.7	1/60	35.7421	35.5344
2	11.65	45	10	15	31455	0	39.9	0.7	1/60	118.3616	117.6739
5	32.48	45	15	15	58464	-	39.9	0.7	1/60	315.4334	325.7637
**No. 2 – Formaldehyde**											
0.5	2.63	0.051	13	15	6.1906	0	39.9	0.7	1/60	0.0308	0.0307
2	8.96	0.051	10	15	21.0905	0	39.9	0.7	1/60	0.0794	0.0789
5	24.29	0.051	13	15	57.1749	0	39.9	0.7	1/60	0.2847	0.2831
**No. 2 – Glutaraldehyde**											
0.5	2.63	0.041	13	15	4.9768	0	39.9	0.7	1/60	0.0248	0.0246
2	8.96	0.041	10	15	22.0416	0	39.9	0.7	1/60	0.0829	0.0825
5	24.29	0.041	13	15	45.9642	0	39.9	0.7	1/60	0.2289	0.2276
**No. 3 – Glutaraldehyde**											
0.5	2.28	0.048	7	15	9.3806	0	39.9	0.7	1/60	0.0243	0.0241
2	8.48	0.048	12	15	20.3520	0	39.9	0.7	1/60	0.0930	0.0925
5	23.22	0.048	15	15	44.5824	-	39.9	0.7	1/60	0.2405	0.2392
10	45.46	0.048	22	15	59.5113	-	39.9	0.7	1/60	0.3211	0.3193
15	76.99	0.048	30	23	73.9104	-	39.9	0.7	1/60	0.5990	0.5957

Supplementary Table 5: Unsteady 1-zone model parameters used for calculations and corresponding results for disinfectant No.4.

Disinfected surface [m²]	Product amount [g]	Weight fraction [%]	Drying time [min]	Exposure time (=Sampling time) [min]	Mass flow during drying time [mg/h]	Mass flow between end of drying time to end of exposure time [mg/h]	Room volume [m³]	Air exchange rate [1/h]	Time increment [h]	$x_{i,0}$ for one time increment shorter than exposure time [mg/m³]	$\bar{x}_i$ for exposure time [mg/m³]
**No. 4 – Hydrogen peroxide**											
0.5	1.78	4.7	2	15	2,509.8000	0	39.9	0.7	1/60	1.8017	1.7912
2	4.35	4.7	5	15	2,453.4000	0	39.9	0.7	1/60	4.4813	4.4553
5	11.57	4.7	8	15	4,078.4250	0	39.9	0.7	1/60	12.1324	12.0619
**No. 4 – Peroxyacetic acid**											
0.5	1.78	0.15	2	15	80.1000	0	39.9	0.7	1/60	0.0575	0.0572
2	4.35	0.15	5	15	78.3000	0	39.9	0.7	1/60	0.1430	0.1422
5	11.57	0.15	8	15	130.1625	0	39.9	0.7	1/60	0.3872	0.3850

Supplementary Table 6: ConsExpo and 2-component model parameters used for Tier 1 and Tier 2 calculations and corresponding results for disinfectants No. 1.

Tier calculation	Room volume [m³]	Air exchange rate [/h]	Product amount [kg]	Weight fraction (%)	Surface area [m²]	Temperature [°C]	Exposure duration [min]	Vapour pressure substance [Pa]	Activity coefficient substance	Molecular weight of substance [g/mol]	Molecular weight of matrix [g/mol]	Vapour pressure Solvent [Pa]	Activity coefficient solvent	Relative humidity [%]	Does area increase?	Application duration [min]	Mass transfer coefficient (substance)[m/h]	Mass transfer coefficient (solvent)[m/h]	ConsExpo MEC [mg/m³]	2-component model MEC [mg/m³]
**No. 1 – Ethanol**																				
1	39.9	0.7	0.00356	45	0.5	21.4	15	5,800	1	46.07	18	2,548.97032	1	37.8	TRUE	0.4	10	10	34.9231	35.2076
2	39.9	0.7	0.00356	45	0.5	21.4	15	42,862	7.39	46.07	18	18,836.8906	7.39	37.8	TRUE	0.4	3.62641996	3.94056607	35.8696	35.9872
1	39.9	0.7	0.01165	45	2	21.5	15	5,800	1	46.07	18	2,564.631	1	43.4	TRUE	2.5	10	10	107.1728	108.7260
2	39.9	0.7	0.01165	45	2	21.5	15	42,862	7.39	46.07	18	18,952.6231	7.39	43.4	TRUE	2.5	2.88548909	6.42790028	109.5249	110.9369
1	39.9	0.7	0.03248	45	5	21.2	15	5,800	1	46.07	18	2,517.89971	1	55.0	TRUE	6.5	10	10	254.0065	262.3219
2	39.9	0.7	0.03248	45	5	21.2	15	42,862	7.39	46.07	18	18,607.2788	7.39	55.0	TRUE	6.5	2.47922 28	5.52287546	261.2789	266.9016

Supplementary Table 7: ConsExpo and 2-component model parameters used for Tier 1 and Tier 2 calculations and corresponding results for disinfectant No. 2.

Tier calculation	Room volume [m^3]	Air exchange rate [/h]	Product amount [kg]	Weight fraction (%)	Surface area [m^2]	Temperature [°C]	Exposure duration [min]	Vapour pressure substance [Pa]	Activity coefficient substance	Molecular weight of substance [g/mol]	Molecular weight of matrix [g/mol]	Vapour pressure Solvent [Pa]	Activity coefficient solvent	Relative humidity [%]	Does area increase?	Application duration [min]	Mass transfer coefficient (substance)[m/h]	Mass transfer coefficient (solvent) [m/h]	ConsExpo MEC [mg/m^3]	2-component model MEC [mg/m^3]
**No. 2 - Formaldehyde**																				
1	39.9	0.7	0.00263	0.051	0.5	21.2	15	430,000	1	30.03	18	2,517.89971	1	45.4	TRUE	0.4	10	10	0.030461981	0.030463135
2	39.9	0.7	0.00263	0.051	0.5	21.2	15	430,000	1	30.03	18	2,517.89971	1	45.4	TRUE	0.4	5.07487457	5.07487457	0.030443709	0.030444834
1	39.9	0.7	0.00896	0.051	2	21.2	15	430,000	1	30.03	18	2,517.89971	1	35.5	TRUE	2.5	10	10	0.096922469	0.096943757
2	39.9	0.7	0.00896	0.051	2	21.2	15	430,000	1	30.03	18	2,517.89971	1	35.5	TRUE	2.5	4.03724787	6.42429316	0.096840839	0.096871225
1	39.9	0.7	0.02429	0.051	5	21.5	15	430,000	1	30.03	18	2,564.631	1	32.7	TRUE	6.5	10	10	0.226690514	0.226825837
2	39.9	0.7	0.02429	0.051	5	21.5	15	430,000	1	30.03	18	2,564.631	1	32.7	TRUE	6.5	3.47271459	5.52597644	0.226377607	0.226599936
**No. 2 – Glutaraldehyde**																				
1	39.9	0.7	0.00263	0.041	0.5	21.2	15	2,300	1	100.12	18	2,517.89971	1	45.4	TRUE	0.4	10	10	0.021546304	0.022715501
2	39.9	0.7	0.00263	0.041	0.5	21.2	15	2,300	1	100.12	18	2,517.89971	1	45.4	TRUE	0.4	2.35714517	2.61406053	0.013679718	0.01697228
1	39.9	0.7	0.00896	0.041	2	21.2	15	2,300	1	100.12	18	2,517.89971	1	35.5	TRUE	2.5	10	10	0.068766254	0.073669678
2	39.9	0.7	0.00896	0.041	2	21.2	15	2,300	1	100.12	18	2,517.89971	1	35.5	TRUE	2.5	1.87519498	6.42429316	0.040029453	0.064982029
1	39.9	0.7	0.02429	0.041	5	21.5	15	2,300	1	100.12	18	2,564.631	1	32.7	TRUE	6.5	10	10	0.15285764	0.175915399
2	39.9	0.7	0.02429	0.041	5	21.5	15	2,300	1	100.12	18	2,564.631	1	32.7	TRUE	6.5	1.61298419	5.52597644	0.073176008	0.142484999

Supplementary Table 8: ConsExpo and 2-component model parameters used for Tier 1 and Tier 2 calculations and corresponding results for disinfectants No. 3.

Tier calculation	Room volume [m³]	Air exchange rate [/h]	Product amount [kg]	Weight fraction (%)	Surface area [m²]	Temperature [°C]	Exposure duration [min]	Vapour pressure substance [Pa]	Activity coefficient substance	Molecular weight of substance [g/mol]	Molecular weight of matrix [g/mol]	Vapour pressure Solvent [Pa]	Activity coefficient solvent	Relative humidity [%]	Does area increase?	Application duration [min]	Mass transfer coefficient (substance)[m/h]	Mass transfer coefficient (solvent) [m/h]	ConsExpo MEC [mg/m³]	2-component model MEC [mg/m³]
**No. 3 - Glutaraldehyde**																				
1	39.9	0.7	0.00228	0.048	0.5	21.5	15	2,300	1	100.12	18	2,564.631	1	34.2	TRUE	0.4	10	10	0.022269396	0.023424747
2	39.9	0.7	0.00228	0.048	0.5	21.5	15	2,300	1	100.12	18	2,564.631	1	34.2	TRUE	0.4	2.35846866	2.61552827	0.014941787	0.018852846
1	39.9	0.7	0.00848	0.048	2	21.7	15	2,300	1	100.12	18	2,596.205	1	35.5	TRUE	2.5	10	10	0.076721184	0.082034998
2	39.9	0.7	0.00848	0.048	2	21.7	15	2,300	1	100.12	18	2,596.205	1	35.5	TRUE	2.5	1.87694967	6.43030461	0.045760712	0.073124245
1	39.9	0.7	0.02322	0.048	5	20.7	15	2,300	1	100.12	18	2,441.66832	1	44.2	TRUE	6.5	10	10	0.172550362	0.195153531
2	39.9	0.7	0.02322	0.048	5	20.7	15	2,300	1	100.12	18	2,441.66832	1	44.2	TRUE	6.5	1.61057005	5.51770575	0.08451401	0.152105585
1	39.9	0.7	0.04546	0.048	10	21.6	15	2,300	1	100.12	18	2,580.376	1	41.7	TRUE	15	10	10	0.20295855	0.25171267
2	39.9	0.7	0.04546	0.048	10	21.6	15	2,300	1	100.12	18	2,580.376	1	41.7	TRUE	15	1.43893567	4.92969781	0.08505069	0.19790236
1	39.9	0.7	0.07699	0.048	15	20.8	24	1,410	1	76.05	18	2,456.751	1	26.7	TRUE	22	10	10	0.37420568	0.45342325
2	39.9	0.7	0.07699	0.048	15	20.8	24	1,410	1	76.05	18	2,456.751	1	26.7	TRUE	22	1.3438042	4.60378373	0.18317503	0.41446254

Supplementary Table 9: ConsExpo and 2-component model parameters used for Tier 1 and Tier 2 calculations and corresponding results for disinfectants No. 4.

Tier calculation	Room volume [m³]	Air exchange rate [/h]	Product amount [kg]	Weight fraction (%)	Surface area [m²]	Temperature [°C]	Exposure duration [min]	Vapour pressure substance [Pa]	Activity coefficient substance	Molecular weight of substance [g/mol]	Molecular weight of matrix [g/mol]	Vapour pressure Solvent [Pa]	Activity coefficient solvent	Relative humidity [%]	Does area increase?	Application duration [min]	Mass transfer coefficient (substance)[m/h]	Mass transfer coefficient (solvent) [m/h]	ConsExpo MEC [mg/m³]	2-component model MEC [mg/m³]
**No. 4 – Hydrogen peroxide**																				
1	39.9	0.7	0.00178	4.70	0.5	19.2	15	214	1	34	18	2,224.95579	1	37.5	TRUE	0.4	10	10	0.774193805	1.666143044
2	39.9	0.7	0.00178	4.70	0.5	19.2	15	64.2	0.3	34	18	2,224.95579	1	37.5	TRUE	0.4	5.89794901	8.04517098	0.181114569	1.432180967
1	39.9	0.7	0.00435	4.70	2	19.8	15	214	1	34	18	2,309.52986	1	33.4	TRUE	2.5	10	10	2.252210013	4.050586704
2	39.9	0.7	0.00435	4.70	2	19.8	15	64.2	0.3	34	18	2,309.52986	1	33.4	TRUE	2.5	4.69732903	6.4074503	0.487804501	3.526834978
1	39.9	0.7	0.01157	4.70	5	20.1	15	214	1	34	18	2,352.86266	1	31.3	TRUE	6.5	10	10	4.410816319	9.639620706
2	39.9	0.7	0.01157	4.70	5	20.1	15	64.2	0.3	34	18	2,352.86266	1	31.3	TRUE	6.5	4.04050391	5.5114998	0.793340134	7.496595529
**No. 4 – Peroxyacetic acid**																				
1	39.9	0.7	0.00178	0.15	0.5	19.2	15	1,410	1	76.05	18	2,224.95579	1	37.5	TRUE	0.4	10	10	0.052643957	0.056785548
2	39.9	0.7	0.00178	0.15	0.5	19.2	15	3,475.65	2.465	76.05	18	2,224.95579	1	37.5	TRUE	0.4	3.16179753	3.92433526	0.050497199	0.053988794
1	39.9	0.7	0.00435	0.15	2	19.8	15	1,410	1	76.05	18	2,309.52986	1	33.4	TRUE	2.5	10	10	0.12560091	0.135019371
2	39.9	0.7	0.00435	0.15	2	19.8	15	3,475.65	2.465	76.05	18	2,309.52986	1	33.4	TRUE	2.5	2.51816407	6.4074503	0.118953402	0.130955405
1	39.9	0.7	0.01157	0.15	5	20.1	15	1,410	1	76.05	18	2,352.86266	1	31.3	TRUE	6.5	10	10	0.277710658	0.314087135
2	39.9	0.7	0.01157	0.15	5	20.1	15	3,475.65	2.465	76.05	18	2,352.86266	1	31.3	TRUE	6.5	2.16605048	5.5114998	0.251861336	0.305053577

Supplementary Table 10: Input parameters for the description of the process, the workplace and the alcoholic wipes (No. 1) for the exposure modelling in Stoffenmanager®.

**Process**	
Process	Surface disinfection with wet tissues
Activity	Handling of liquids where only small amounts of product may be released
Activity in breathing zone	Yes
Multiple employees	No
Evaporation, drying or curing after activity	Yes
Respiratory protection	No protection
Process temperature	20 °C or 293.15 K
**Workplace**	
Workplace	Test chamber at the IFA
Volume of the working room	Volume under 100 m^3
Ventilation working room	No general ventilation
Regular cleaning of work area	Yes
Regular inspection and maintenance	Yes
Control measures at the source	No control measures at the source
Segregation of employee	The employee does not work in a cabin
**No. 1 Alcoholic wipes***	
Dilution of the product (as % of product)	100% product, 0% water
Component	Ethanol
Vapour pressure component	5800 Pa (20 °C or 293.15 K)
Concentration in initial product	45%

* The applied disinfectant was described according to its safety data sheet.

Supplementary Table 11: Comparison of the modelled values (PRED) of the unsteady 1-zone model, of ConsExpo and of the 2-component-model with the mean of the measured values from stationary air sampling (EXP).

**No.**	**Disinfectant active ingredient**	**Disinfected surface [m^2]**	**Unsteady 1-zone model**	**ConsExpo (Tier 1)**	**ConsExpo (Tier 2)**	**2-component model (Tier 1)**	**2-component model (Tier 2)**
			**Comparison (PRED/EXP $_{\text{stationary air sampling}}$)**				
**1**	Ethanol	0.5	0.56	0.55	0.56	0.55	0.56
		2	1.00	0.92	0.94	0.93	0.95
		5	1.16	0.91	0.93	0.94	0.95

Supplementary Table 12: Mean values of measured data from personal air sampling and modelled values with the unsteady 1-zone model under different ventilation conditions (λ = 0.7/h, 0.8/h or 0.9/h) as well as the comparison of the modelled values (PRED) with the mean of the measured values from personal air sampling (EXP personal air sampling).

**No.**	**Disinfectant active ingredient**	**Disinfected surface [m²]**	**Mean value (EXP) [mg/m³] (Personal air sampling)**	**Modelled values (PRED) [mg/m³] (Unsteady 1-zone model)**			**Comparison (PRED/EXP $_{personal\ air\ sampling}$)***		
				**λ = 0.7/h**	**λ = 0.8/h**	**λ = 0.9/h**	**λ = 0.7/h**	**λ = 0.8/h**	**λ = 0.9/h**
**1**	Ethanol	0.5	81	35.5344	31.0926	27.6378	0.44	0.38	0.34
		2	167	117.6739	102.9646	91.5241	0.7	0.62	0.55
		5	380	325.7637	285.0432	253.3718	0.86	0.75	0.67
**2**	Formaldehyde	0.5	0.024	0.0307	0.0268	0.0238	1.28	1.12	0.99
		2	0.051	0.0789	0.0690	0.0614	1.55	1.35	1.20
		5	0.173	0.2831	0.2477	0.2202	1.64	1.43	1.27
	Glutaraldehyde	0.5	n. d.	0.0246	0.0216	0.0192	-	-	-
		2	n. d.	0.0825	0.0722	0.0641	-	-	-
		5	0.052	0.2276	0.1991	0.1770	4.38	3.83	3.40
**3**	Glutaraldehyde	0.5	n. d.	0.0241	0.0211	0.0188	-	-	-
		2	n. d.	0.0925	0.0809	0.0719	-	-	-
		5	0.047	0.2392	0.2093	0.1860	5.09	4.45	3.96
		10	0.055	0.3193	0.2794	0.2483	5.81	5.08	4.51
		15	0.089	0.5957	0.5212	0.4633	6.69	5.86	5.21
**4**	Hydrogen peroxide	0.5	0.39	1.7912	1.5673	1.3932	4.59	4.02	3.57
		2	1.26	4.4553	3.8984	3.4652	3.53	3.09	2.75
		5	2.48	12.0619	10.5542	9.3815	4.86	4.26	3.78
	Peroxyacetic acid	0.5	n. d.	0.0572	0.0500	0.0445	-	-	-
		2	0.09	0.1422	0.1244	0.1106	1.58	1.38	1.23
		5	0.21	0.3850	0.3368	0.2994	1.83	1.60	1.43

n. d. = Not determinable, i.e., for glutaraldehyde the concentration is below the analytical detection limit of 0.041 mg/m³ and for peroxyacetic acid below 0.05 mg/m³.
* In the case, that the mean value of the personal air sampling was not determinable (n.d.), no PRED/EXP could be determined.

Supplementary Figure 1: Graphical presentation of the modelled exposure distribution by Stoffenmanager® for ethanol (No. 1).

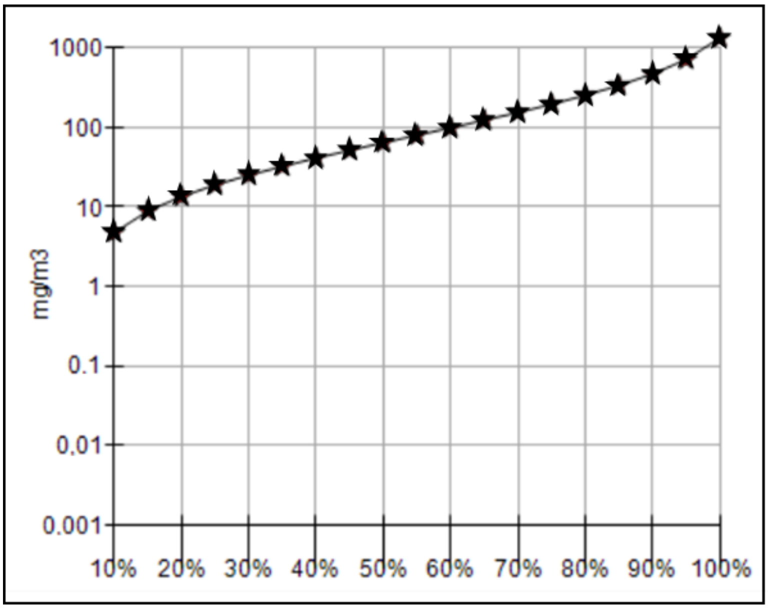

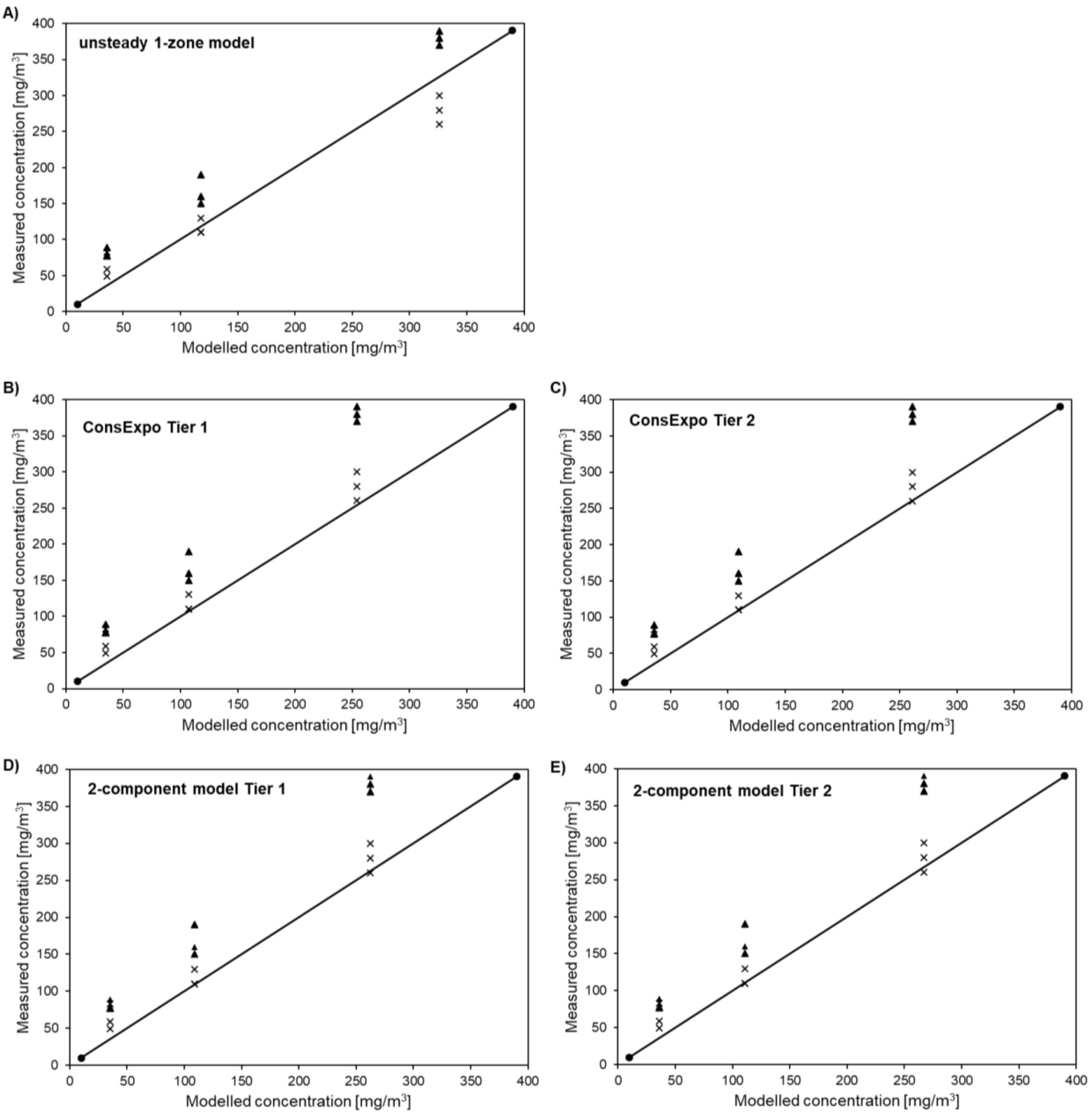

Supplementary Figure 2: Measured data from ethanol (No. 1) of personal air sampling (▲) and stationary air sampling (**x**) plotted against the modelled data of A) the unsteady 1-zone model, B) ConsExpo (Tier 1 calculation), C) ConsExpo (Tier 2 calculation), D) 2-component model (Tier 1 calculation) and E) 2-component model (Tier 2 calculation). Each scatter plot includes data for the three different sizes of disinfected surface.

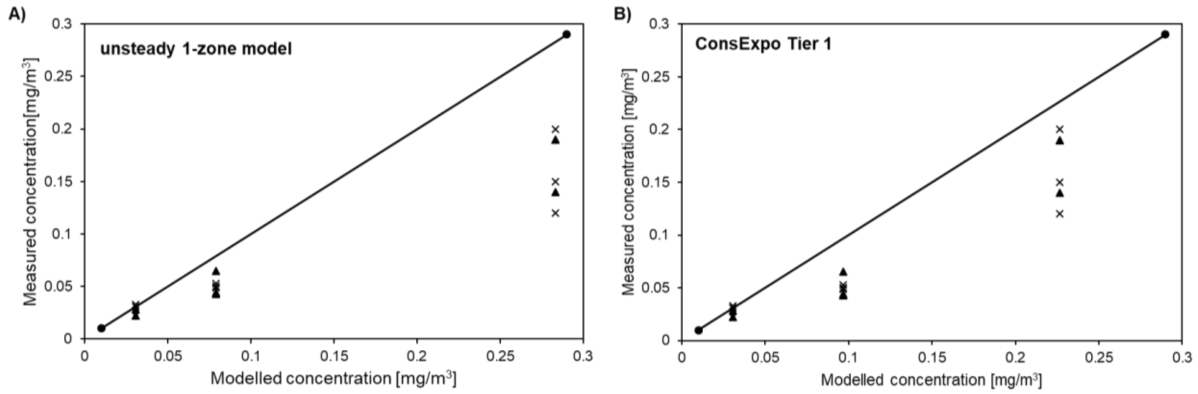

Supplementary Figure 3: Measured data from formaldehyde (No. 2) of personal air sampling (▲) and stationary air sampling (**x**) plotted against the modelled data of A) the unsteady 1-zone model and B) ConsExpo (Tier 1 calculation). Scatter plots of ConsExpo (Tier 2 calculation), as well as 2-component model (Tier 1 and 2 calculation) are not further shown because the modelled values are nearly identical. Each scatter plot includes data for the three different sizes of disinfected surface.

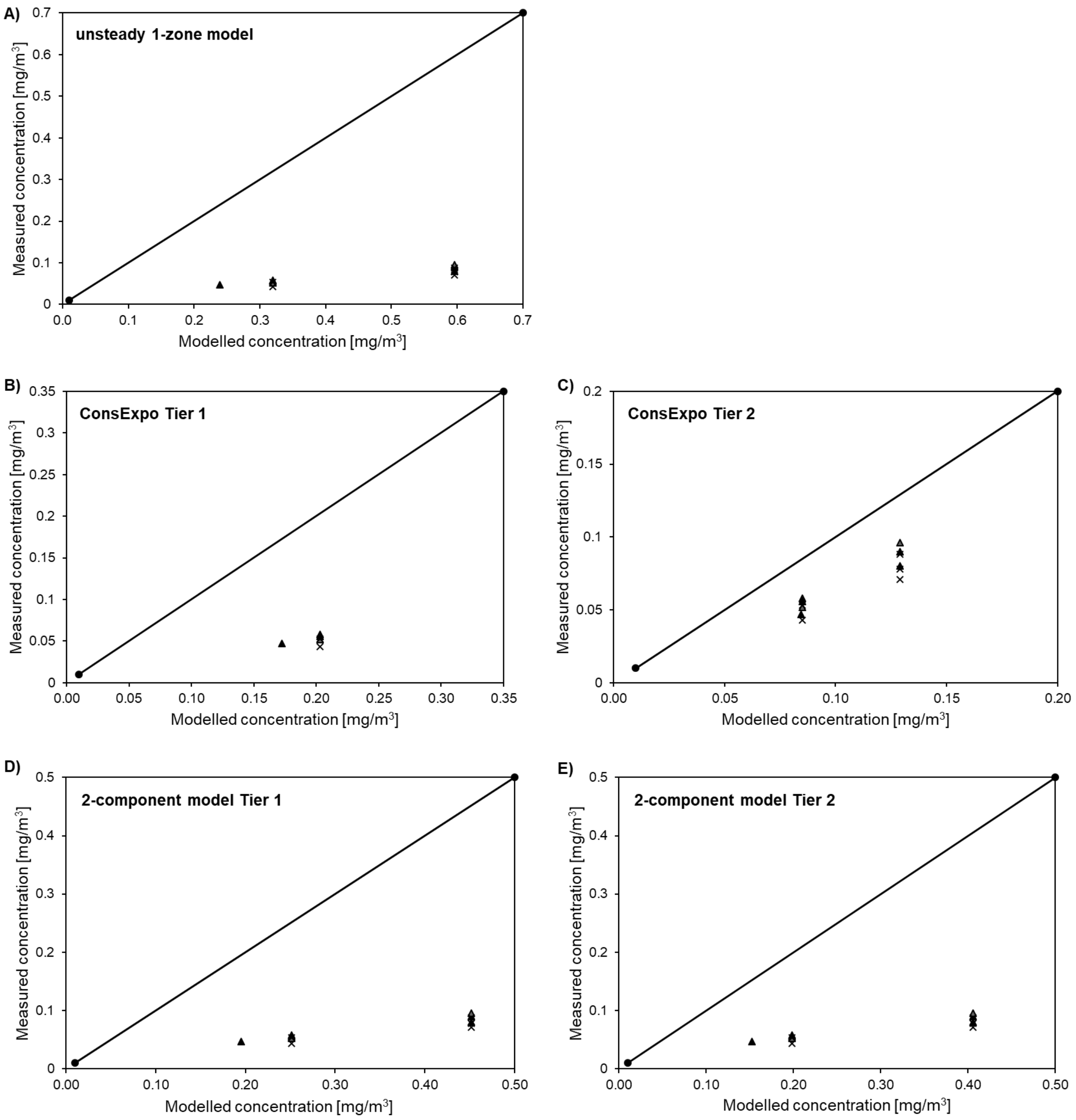

Supplementary Figure 4: Measured data from glutaraldehyde (No. 3) of personal air sampling (▲) and stationary air sampling (**x**) plotted against the modelled data of A) the unsteady 1-zone model, B) ConsExpo (Tier 1 calculation), C) ConsExpo (Tier 2 calculation), D) 2-component model (Tier 1 calculation) and E) 2-component model (Tier 2 calculation). Each scatter plot includes data for the three different sizes of disinfected surface.

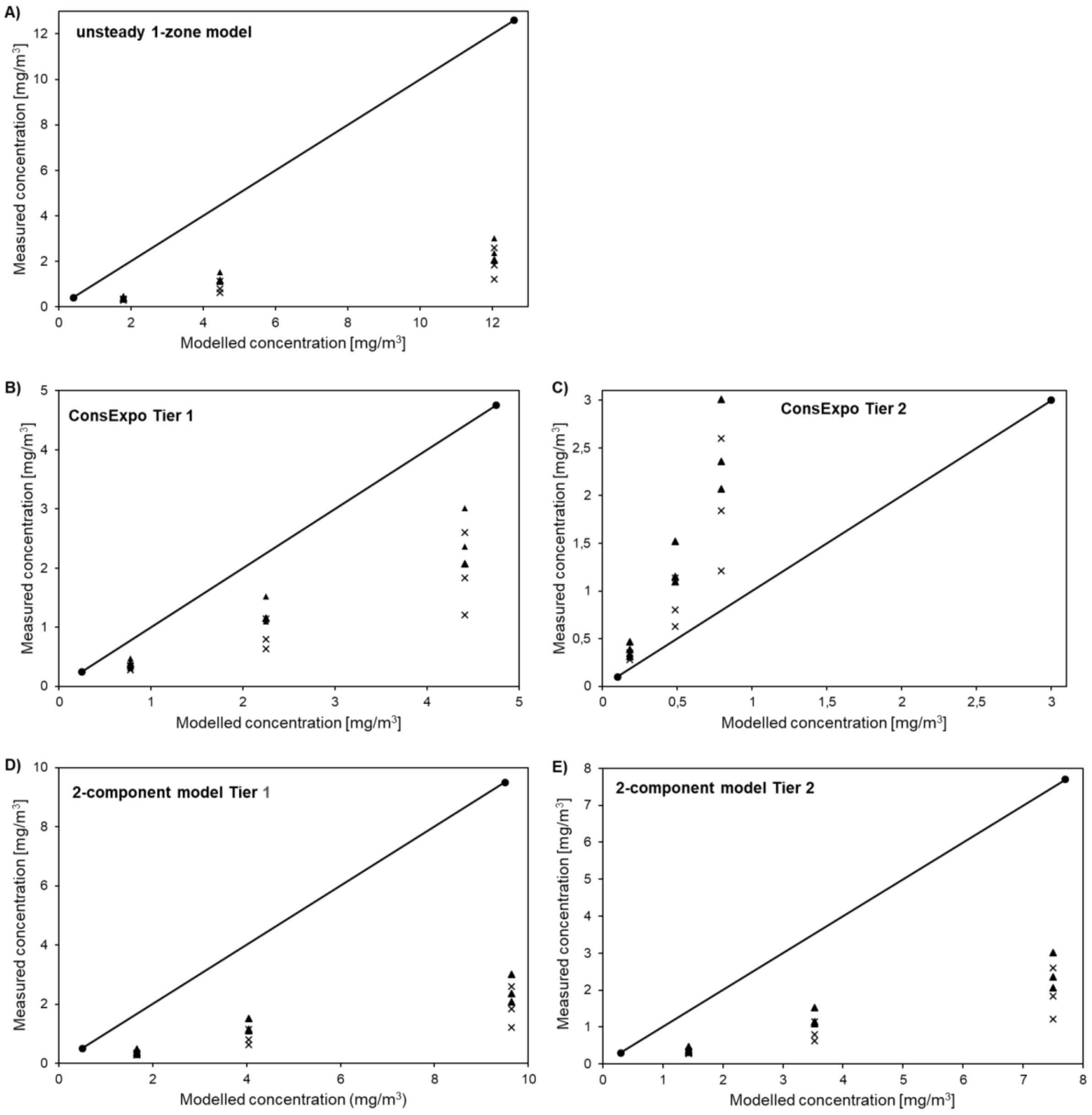

Supplementary Figure 5: Measured data from hydrogen peroxide (No. 4) of personal air sampling (▲) and stationary air sampling (**x**) plotted against the modelled data of A) the unsteady 1-zone model, B) ConsExpo (Tier 1 calculation), C) ConsExpo (Tier 2 calculation), D) 2-component model (Tier 1 calculation) and E) 2-component model (Tier 2 calculation). Each scatter plot includes data for the three different sizes of disinfected surface.

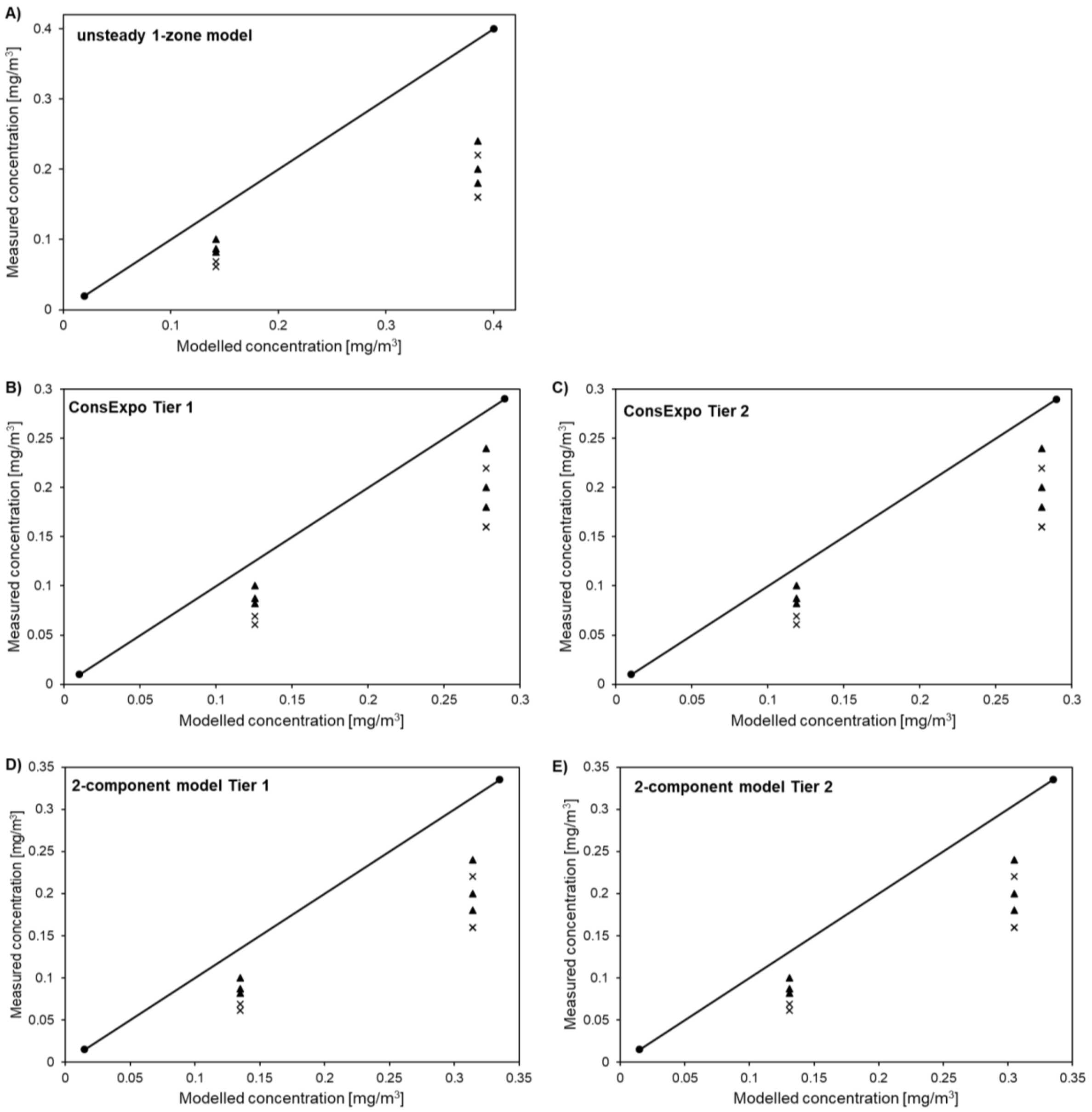

Supplementary Figure 6: Measured data from peroxyacetic acid (No. 4) of personal air sampling (▲) and stationary air sampling (x) plotted against the modelled data of A) the unsteady 1-zone model, B) ConsExpo (Tier 1 calculation), C) ConsExpo (Tier 2 calculation), D) 2-component model (Tier 1 calculation) and E) 2-component model (Tier 2 calculation). Each scatter plot includes data for the three different sizes of disinfected surface.
